# Cooperative Opportunistic Pressure Based Routing for Underwater Wireless Sensor Networks

**DOI:** 10.3390/s17030629

**Published:** 2017-03-19

**Authors:** Nadeem Javaid, Muhammad, Arshad Sher, Wadood Abdul, Iftikhar Azim Niaz, Ahmad Almogren, Atif Alamri

**Affiliations:** 1 COMSATS Institute of Information Technology, Islamabad 44000, Pakistan; muhammadnasiri@yahoo.com (M.); arshadsher92@gmail.com (A.S.); ianiaz@comsats.edu.pk (I.A.N.); 2 Research Chair of Pervasive and Mobile Computing, College of Computer and Information Sciences, King Saud University, Riyadh 11633, Saudi Arabia; aabdulwaheed@ksu.edu.sa (W.A.); ahalmogren@ksu.edu.sa (A.A.); atif@ksu.edu.sa (A.A.)

**Keywords:** underwater wireless sensor network, energy balancing, cooperation, opportunistic routing

## Abstract

In this paper, three opportunistic pressure based routing techniques for underwater wireless sensor networks (UWSNs) are proposed. The first one is the cooperative opportunistic pressure based routing protocol (Co-Hydrocast), second technique is the improved Hydrocast (improved-Hydrocast), and third one is the cooperative improved Hydrocast (Co-improved Hydrocast). In order to minimize lengthy routing paths between the source and the destination and to avoid void holes at the sparse networks, sensor nodes are deployed at different strategic locations. The deployment of sensor nodes at strategic locations assure the maximum monitoring of the network field. To conserve the energy consumption and minimize the number of hops, greedy algorithm is used to transmit data packets from the source to the destination. Moreover, the opportunistic routing is also exploited to avoid void regions by making backward transmissions to find reliable path towards the destination in the network. The relay cooperation mechanism is used for reliable data packet delivery, when signal to noise ratio (SNR) of the received signal is not within the predefined threshold then the maximal ratio combining (MRC) is used as a diversity technique to improve the SNR of the received signals at the destination. Extensive simulations validate that our schemes perform better in terms of packet delivery ratio and energy consumption than the existing technique; Hydrocast.

## 1. Introduction

Recently, UWSNs have gained much attention due to high demand of these networks in many applications. Like prediction of natural disasters, security, measurement of water traits, exploration of underwater world, pollution control, exploration of natural resources in underwater, etc. However, unique characteristics of the aquatic environment have posed many challenges for underwater communication [1], like low bandwidth, high propagation delay, path loss, high bit error rate (BER), etc. resulting in high energy consumption, low reliability of data packet delivery, etc. [2].

**Figure 1:**
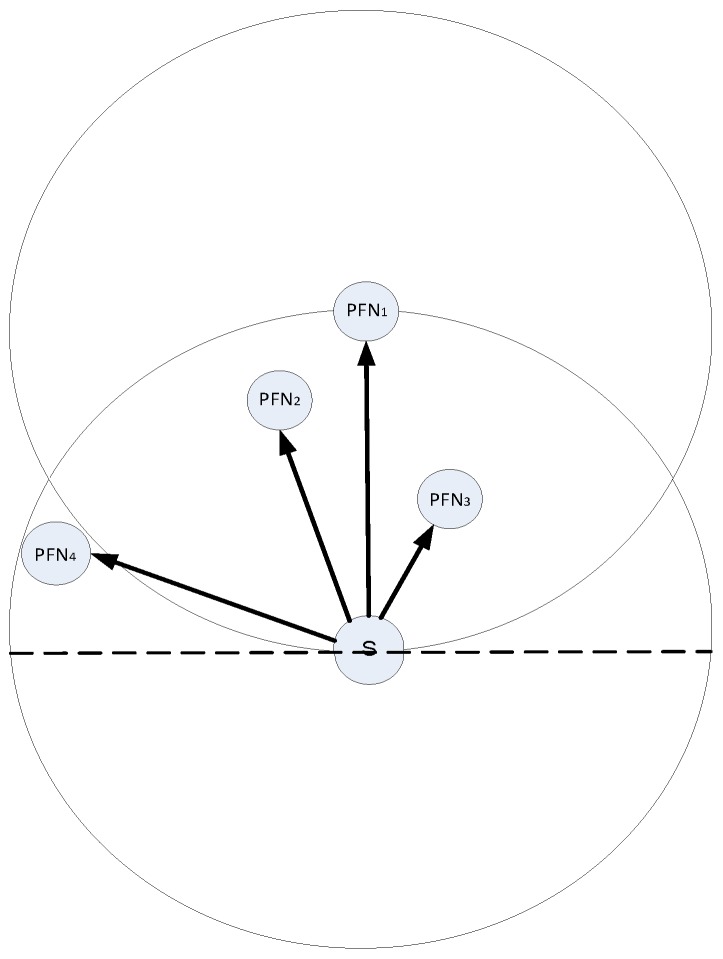
How duplicate packets are transmitted in opportunistic routing.

In such conditions, opportunistic routing is a remarkable technique in sensor networks due to its redundant transmissions. However, the reliability is achieved by redundant packet transmission that is one of the influential factor on the performance of the opportunistic routing. As shown in Figure 1, let a source node (S) broadcasts a data packet to its neighbor nodes ($PFN_{1}$, $PFN_{2}$, $PFN_{3}$, $PFN_{4}$), the $PFN_{1}$ has highest priority because it is deployed near the sink. It will forward a data packet and acknowledge its neighbor nodes in its transmission range $(PFN_{2}$, $PFN_{3})$. The $PFN_{4}$ will not overhear the acknowledgement from $PFN_{1}$ because it is deployed outside its transmission range. Therefore, after its specific holding time, it will consider that the data packet is not forwarded and it broadcasts the data packet resulting in redundant transmission and unnecessary energy will be consumed [3].

Although some pressure based routing protocols for underwater sensor networks [4] take advantage of the redundant transmissions to recover the data packets from void holes. Similarly, in [5], the authors proposed analysis of the cooperative routing with incremental best relay scheme. In this scheme, a single bit message received from a node that indicates whether the data packet transmitted directly, received successfully or not. If the data packet is not within the predefined threshold then a relay node broadcasts the data packet which is combined at the destination to obtain the error free packet. Authors [6] presented void aware pressure routing (VARP) algorithm to propagate periodic beacon messages in the entire network for establishing paths to the nearest sink. The data packets are relayed via multiple paths to the destination for reliable data delivery. Similarly, the authors in [7] suggested an energy efficient cooperative scheme for the selection of the best relay node amongst the successful candidates at each hop those received the data packet successfully.

However, limitations of [4,5,6,7] are: (1) long routing paths lead to the path loss and high propagation delay; (2) They are efficient in dense network conditions however when the network sparsity increases, the length of routing paths increases that increase the breakage probability of the path from the source to the destination resulting in high packet drop ratio. Therefore, Co-Hydrocast, improved Hydrocast and Co-improved Hydrocast opportunistic based routing protocols are proposed in this paper. These protocols are presented to increase the network throughput and reliability of the data transmissions in the dynamic nature of the aquatic environment by shortening the recovery path between the source and the destination. These routing techniques avoid void node occurrence by obtaining neighbors of a next relay node up to two hops. Each forwarding node selects its best relay node having at least one neighbor and minimum distance relative to the sink. The redundant transmissions are avoided while ensuring that each candidate node listens to each other before transmitting a data packet towards the destination that results in minimum energy consumption and high network throughput.

The rest of the paper is organised as follows: In Section 2 related work is presented. Details of our proposed schemes are discussed in Section 3. Section 4 validates the performance through simulation results of our proposed techniques. Trade-offs are given in Section 5. Finally Section 6 concludes our proposed work by discussing the findings of our proposed schemes.

## 2. Related Work

Many routing protocols are proposed for energy efficiency in UWSNs. Like depth based routing protocol (DBR) [8] uses depth only as a routing metric for data packet forwarding from a source node to a destination. In this protocol, a node with lowest depth from the source node with respect to sink is selected as a next hop forwarder. This process repeats until the packet reaches the destination. The packet forwarding decision is made locally at each hop to select a best next hop forwarder. It performs better at dense network in terms of network throughput and consumes comparatively less energy. However, in sparse deployment, the breakage probability of a path between the source and the destination increases due to dynamic nature of underwater environment. Also lengthy paths results in more energy consumption and low packet delivery ratio. Moreover, the chances of void hole occurrence increases.

The authors present an energy efficient depth based routing protocol (EEDBR) in [9] that considers both residual energy and depth of a sensor node as routing metrics for data forwarding. Like DBR, EEDBR also forwards data packets towards the sink using greedy approach. Before transmitting the data packet, a source node looks for a next hop forwarder that has high residual energy from his neighbor nodes and lowest depth. The EEDBR achieves energy efficiency and network throughput comparatively higher than the DBR. However, limitations still exists that void nodes occurrence increases when network becomes more sparse resulting in high energy consumption on sensor nodes near the sink and degrades the network performance.

In [10], the authors present a weighting depth and forwarding area division DBR (WDFAD-DBR) routing protocol for UWSNs. It makes the routing decisions based on the sum of the depth difference between two hops (depth difference from current node to next neighbor node and from next neighbor node to its neighbor node) to avoid the void holes. WDAFD-DBR uses the reuleaux triangle to ensure that every neighbor node overhear the data transmission from a high priority neighbor node for eliminating the redundant transmissions to conserve the network energy. The neighbor node priority is defined on the basis of depth from the sink which is used for calculating a holding time of low priority sensor nodes to suppress the transmission for specific time. This scheme achieves reliable data delivery in sparse conditions, less energy consumption by dividing the forwarding area, and end to end latency is also minimized.

Ayaz et al. [11] propose a distributed routing protocol called hop by hop dynamic addressing based routing (H2-DAB). Each Node maintains two types of IDs called node ID and hop ID based on its distance from the sink. A sensor node that has the lowest distance, gets the smallest hop ID and vice versa that helps the sender node in nominating a next hop forwarder. It does not requires dimensional information, however, H2-DAB utilizes the multiple sink architecture to enhance the packet delivery ratio at the cost of high propagational delay and maximal energy consumption.

Yoon et al. [12] improve the network lifetime by using AUVs (Autonomous Underwater Vehicles) as relay nodes for collecting data packets from sensor nodes. After collection of packets, these AUVs transmit the data to the sink. This routing protocol is proposed for large scale networks to minimize the energy consumption and enhance the data delivery ratio. It minimizes long data paths by using AUVs as relay nodes on elliptical paths to gather data packets from sensor nodes directly and via multi-hopping. However, it achieves high network throughput and optimal network lifetime at the cost of end to end delay.

Authors in [13] present an energy efficient routing protocol that choose a next forwarder node having high link quality, minimum number of hops, and high residual energy among his neighbor nodes. It spread the data traffic from a high residual energy node to low residual energy nodes to balance the energy consumption across the network for enhancing the network lifetime. The data packet forwarding decisions are taken locally on the basis of shortest path between the sender and the receiver. This scheme achieves optimal network lifetime by balancing the energy in the network.

In [6], the authors introduce void aware pressure routing protocol (VARP) in UWSNs. In entire network, periodic beacon messages are propagated which are used to set up next hop forwarders and establish routing paths to the nearest sink. It uses the directional opportunistic forwarding mechanism to forward a data between the source and the destination. With the use of directional forwarding, data packets are delivered even in the presence of void nodes because at each hop, it is checked that if next node is a void then it changes the direction of the forwarding data, otherwise same routing path is used that established via beacon message, to send the data towards the destination.

Geographic and opportunistic routing for UWSNs (GEDAR) is presented in [2]. It is an any-cast routing protocol in which sensor nodes broadcast data packets to multiple sinks in their transmission range. When a source node finds no neighbor in its vicinity then it looks for the recovery procedure to adjust the depth of a sensor node in order to find forwarder node. During the data forwarding, at each hop sensor nodes propagate beacon messages to locate a next forwarder node with minimum distance than the current node with respect to the sink. It takes advantage of the opportunistic routing redundant transmissions for reliable data packet delivery at the destination. GEDAR achieves high packet delivery ratio, avoids void holes, and also provides the recovery mechanism (depth adjustment) to recover data from a void region.

Taking advantage from the broadcast nature of wireless sensor networks, authors in [7] present a novel scheme for cooperative communication calls energy-efficient cooperative communication scheme (EECC). At each transmission, a best node is selected as a cooperative relay from the set of nodes which successfully receive the transmitted data packet.

In [14], an efficient routing protocol is presented that uses mobile sinks and cooperative routing. Nodes select their relays and destination nodes based upon their depth information. Data packets are directly transmitted from sender nodes to the receiver nodes and also via relay nodes. Destination nodes after combining both copies of the same signal, transmits the combined signal to the mobile sink. Different mobile sinks at different depths, collect data packets from destination nodes, to improve the network throughput and reduce the packet drop ratio. However, the message exchange overhead is high resulting in high energy consumption of the network.

In [15], a time of arrival ranging mechanism is introduced to limit the redundant transmissions issue in DBR routing protocol, and two different techniques are proposed: the first scheme focuses on energy efficiency, while the second scheme is presented for delay intolerant applications. In the former scheme, a holding time is computed on the basis of depth from the sink and only high priority node transmits the data packet and acknowledge the other neighbor nodes to drop the respective packet. It performs better in terms of energy consumption by avoiding the redundant packet transmission at the destination. In later one, the end to end delay is minimized at the cost of energy.

Fatemeh et al. [16] propose a scheme for reducing collision probability by combining route selection, cooperative transmission and optimal power allocation. This algorithm chooses a path with less collision probability for forwarding data packets. The path with less collision is selected by cooperative transmission and power allocation to minimize the collision and that helps to restrict the collision outage probability within the predefined threshold. This mechanism is repeated at each link until the data packet reaches the sink. It proves that to minimize the collision probability, collision awareness at a link is very essential to enhance the network performance.

Authors in [5] introduce performance analysis of cooperative routing with incremental best relay technique. In this technique, a single bit message response from the receiving node indicates whether the direct transmitted packet received successfully or not. In case of failure, optimum relay forwards the received signal to the destination. However, in this scheme high packet drop ratio is the main constraint.

A scheme for maximizing the network lifetime using mobile nodes is introduced in [17]. This scheme is designed particularly for applications in which delay can be tolerated. Due to no time bound for data transfer in case of random mobility of the mobile sink results in data overflow from the mobile sink buffer and leading to high packets drop rate.

Authors in [18] present a balanced energy consumption routing technique in UWSNs. They use a static underwater sink, while sensor nodes move around the sink at low speed. An optimum energy level is defined to balance the energy consumption in the entire network. It consists of two phases: route establishment phase and balanced data transmission phase. In route setup phase, relay nodes are selected those have minimum distance from the sink and a cost function is also computed at each hop to select a best next forwarder node. Whereas in data transmission phase, residual energy of transmitting node is divided in to *n* levels, if next node have higher residual energy from that particular level of source node is selected as a next hop, this process repeats until a data packet is delivered to the destination.

In [19], a cooperative transmission scheme is presented for a clustered network. In this scheme, a cluster head broadcasts data packet in two phases; inter-cluster and intra-cluster. In the first phase, the cluster head transmits the data packet with low transmission power, and all sensor nodes within the cluster that successfully decode the data packet are selected as relay nodes. In the second phase; the cluster head directly transmits the data packet and also via all selected relays to the neighboring cluster heads. However, if the network density is high and each cluster includes high number of sensor nodes, then the energy consumption of the network increases.

Authors in [20] propose two energy balanced routing protocols for UWSNs. The first one is, an efficient and balanced energy consumption technique (EBET), it avoids direct transmission over long distances by keeping the direct transmissions at each hop within or equal to the optimum distance threshold. In the second technique, an enhanced EBET (EEBET), entire network energy is balanced by considering depth threshold that balances the energy within the sectors to prolong the network lifetime. However, the prolongation of the network is achieved in both schemes at the cost of propagational delay. Summary of the related work is shown in Table 1.

## 3. Our Proposed Work

**Figure 2:**
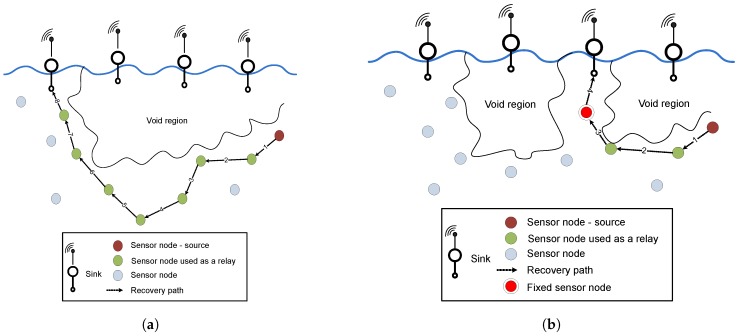
Recovery paths in existing and proposed schemes. (**a**) Long recovery path for data packet delivery; (**b**) Short recovery path due to fixed node.

In Hydrocast [4], the authors introduce a recovery mechanism called lower depth first method, for routing data packets from void nodes. Data packet drop issue is minimized to some extent via this recovery method and the protocol shows good performance when the network density is high. However, long recovery paths are established for data packet delivery from void nodes in the sparse network conditions as shown in Figure 2a. When the network density decreases, the probability of a lengthy recovery path establishment increases, due to which many nodes are involved in forwarding data packets to the nearest sink. This problem results in high average end-to-end latency and high energy consumption in the network.

We use the opportunistic routing technique to enhance the network throughput and also exploit the strategic locations in the network for the deployment of nodes to minimize the average propagation delay. In this paper, three opportunistic routing techniques are proposed: Improved Hydrocast, Co-Hydrocast, and Co-Improved Hydrocast. Details are given in the following subsections.

### 3.1. Forwarding Set Selection

**Figure 3:**
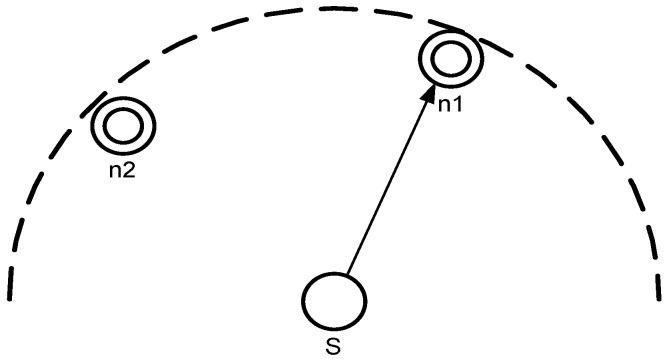
Neighbor node of lower pressure level has high priority to be selected as the next hop forwarder.

The mechanism of a forwarder set is same for all the schemes (Improved Hydrocast, Co-Hydrocast, and Co-Improved Hydrocast). In the proposed schemes, sensor nodes are assumed static [21,22], all nodes find their neighbors and sub neighbors, and calculate their Euclidean distance with neighbors. Suppose that a source node has a set of neighbors $\Gamma_{k}$ in which *k* neighbors are sorted based on their progress towards the destination and the packet delivery probability [23]. The priority is given to nodes that are closed to the sink with at least one neighbor as shown in Figure 3 that source node *S* has two lower pressure level neighbor nodes $n_{1}$ and $n_{2}$. $n_{1}$ has lower pressure level and it is closer to the destination, so it is selected as a next hop forwarder and calculated according to Equation (1).

(1) $$\begin{array}{c} \begin{array}{c} X_{i}=x_{o}+R_{i}\cos\left(\theta \right)\\ Y_{i}=y_{o}+R_{i}\sin\left(\theta \right) \end{array} \end{array}$$

Where *θ* varies between 0–180 degree for forwarder selection, $x_{o}$ and $y_{o}$ shows the origin of the source node, $R_{i}$ is the radius of transmission range and $X_{i}$ and $Y_{i}$ are the co-ordinates of the next forwarder nodes.

**Figure 4:**
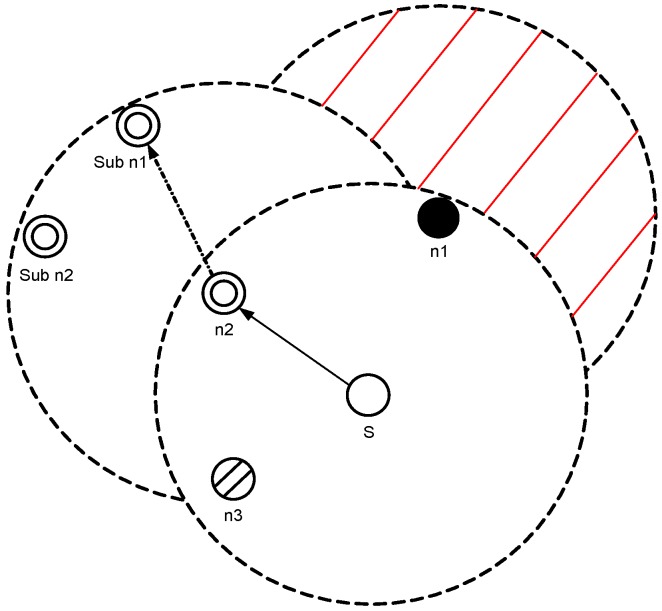
Next hop forwarder selection.

A void node is not selected as a forwarder as shown in Figure 4, S has three neighboring nodes $n_{1}$, $n_{2}$ and $n_{3}$ within its transmission range. $n_{1}$ is a void node, and $n_{3}$ has higher pressure level than *S*. $n_{2}$ is not a void node and it has two neighbors (sub $n_{1}$ and sub $n_{2}$). $n_{2}$ also has lower pressure level than *S*, therefore $n_{2}$ has the highest priority to be chosen as a forwarder. The expected packet advancement (EPA) [24] is given in Equation (2).

(2) $$EPA\left(\Gamma_{k}\right)=\sum_{i=1}^{k}d_{n_{i}}^{p}p_{n_{i}}\prod_{j=0}^{i-1}\left(1-p_{n_{i}}\right)$$

(3) $$p_{n_{i}}=\sum_{i=1}^{k}\frac{1}{n_{neigh}}$$

where $n_{neigh}$ denotes the number of neighbors of the $S_{i}$, higher the number of neighbors greater is the probability of packet advancement. Suppose we have one neighbor and its sub neighbors are zero then the probability of a packet to reach its destination is zero according to Equation (2). Whereas in other case, when neighbor nodes are greater than one, then the probability of packet delivery is high and so on. Even if a *S* neighbor nodes with same number of sub neighbor nodes then the decision is made on the basis of low pressure than *S*. Where $d_{n_{i}}^{p}$ is the $n_{i}$ neighbor progress towards the destination (in meters), and $p_{n_{i}}$ is the $n_{i}$ neighbor packet delivery probability. The neighbor with the highest priority becomes the next hop forwarder and if it fails to transmit the data packet, then a next neighbor with high priority contributes to deliver the data $d_{n_{i+1}}^{p}p_{n_{i+1}}\left(1-p_{n_{i+1}}\right)$. The average holding time (AHT) for a candidate node is calculated as given in Equation (4).

(4) $$AHT=\frac{W_{f}\times H_{t}}{speed\times PL}$$

$W_{f}$ is computed according to the Equation (5).

(5) $$W_{f}=P_{level}\times n_{neigh}$$

where $H_{t}$ is a holding time, speed is the acoustic channel speed (1500 m/s), $W_{f}$ denotes the weight function, $P_{level}$ represents a pressure level of a node, and PL is a path loss between the source and the destination.

**Figure 5:**
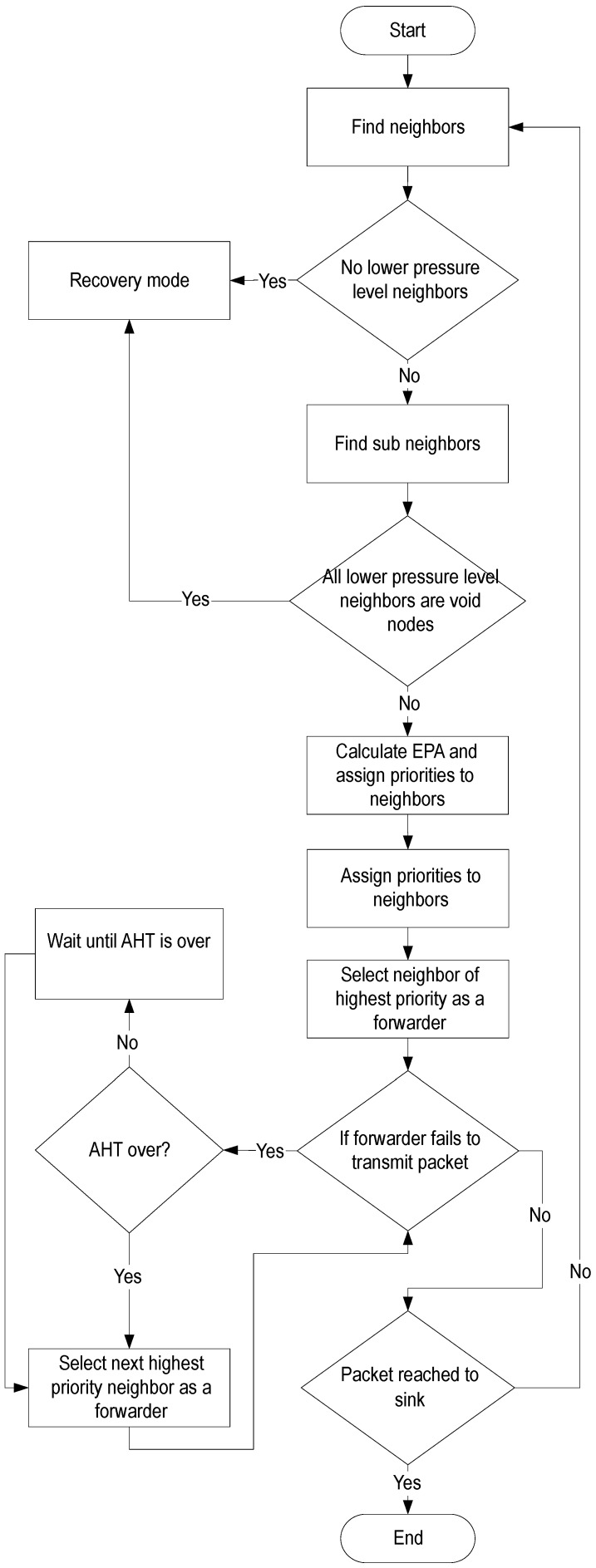
Forwarding set selection.

When a node transmits a data packet, all neighbors those receive the data packet, set a holding time according to Equation (4). A priority is given to a neighbor node which is not a void node and has a less holding time. The selected forwarder node transmits the received data packet, if the received data packet SNR is not within a predefined threshold then nodes start cooperation that is discussed in Section 3.5. A node with the second highest priority acts as a relay node in the cooperation process which is discussed in Section 3.4. Figure 5 shows the forwarding mechanism, if a source node is itself a void node, or all lower pressure level neighbor nodes are void nodes, then the transmission shifts to recovery mode and the source node selects a higher pressure level neighbor as the next hop forwarder, as shown in Figure 5 (recovery mode is discussed in Section 3.2).

### 3.2. Recovery Mode

**Figure 6:**
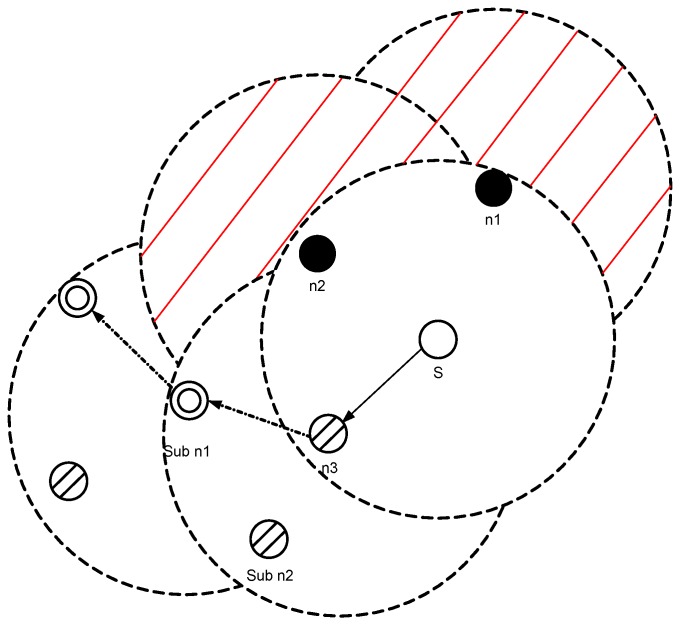
Higher pressure level neighbor is selected as forwarder incase all lower pressure level neighbors are in void region.

When a *S* is unable to find lower pressure node then all proposed algorithms (Improved Hydrocast, Co-Hydrocast, and Co-Improved Hydrocast) follow the same void node recovery mechanism. Let a *S* has to transmit a data packet and it has no neighbor node in the transmission range. It wastes a lot of energy, therefore to avoid this problem, backward transmission mechanism is adopted to find a path to the final destination. The procedure is discussed below:

Figure 6 shows case 1, when a source node *S* has only void nodes as lower pressure nodes as neighbors. n1, n2 and n3 are the neighbors of *S*. Both n1 and n2 have lower pressure level than S, and both are void nodes. n3 has higher pressure level than *S* and is not a void node. So *S* selects n3 as a forwarder and transmits the data packet to it. This backward transmission is changed to greedy forwarding as soon as a lower pressure level neighbor node is found that it is not in a void region. The backward transmission starts when there is no neighbor node which is computed according to Equation (1) and *θ* varies from 180 to 360 degree to find out a lower level neighbor that is not a void node as shown in Figure 6. It ends when n3 finds a neighbor (Sub n1) that has a lower pressure level neighbor than itself, and is not a void node. In this way loops are avoided during the packet forwarding process.

In case 2, a *S* selects a higher pressure level neighbor as the next hop forwarder if it has no lower pressure level neighbors then backward transmission ends as soon as a lower pressure level neighbor is found. In this way, average throughput of the network is increased.

### 3.3. Role of Fixed Deployed Nodes

The mechanism of backward transmissions is avoiding the void region however, it establishes lengthy recovery paths resulting in high energy consumption due to the involvement of more number of hops as shown in Figure 2a. To counter this problem, the strategic deployment of fixed nodes is adopted in Improved Hydrocast and Co-Improved Hydrocast for minimizing number of transmissions. The details are discussed below:

Let *n* nodes be deployed at different strategic locations at sparse conditions. The objectives of strategic location is maximum coverage of the network and avoidance of void regions when the network becomes sparse. These strategically deployed nodes find sparse regions with the objective to the reduce number of transmissions for delivering a data packet as shown in Figure 2b. The strategic locations are chosen according to Equation (6) as follows:

(6) $$TS_{Region}=\sum_{S_{reg=1}}^{S_{n}}\sum_{N=1}^{N}S_{region}\left(S_{n},N\right)$$

where $TS_{Region}$ shows the total number of sparse regions, $S_{reg}$ is a region in which *N* are less and also they can not transmit data packets towards the destination. $S_{reg}$ shows the index of the sparse region with the index of number of nodes *N*. The number of strategic nodes depends on the number of $S_{reg}$. More the $S_{reg}$, greater number of strategic nodes are required to avoid lengthy recovery paths. The nodes that are deployed at strategic locations move in sparse networks with the speed of 0.3 m/s in one scenario discussed in simulation Section 4 and 7.71 m/s in other scenario in order to reduce the distance between the source and destination in sparse networks. The mobility of sensor nodes at strategic locations guaranteed the maximum coverage of the network. Strategic locations are computed according to Equation (7).

(7) $$SL_{node}=\sum_{SL_{node=1}}^{SL_{n}}\sum_{N=1}^{N}S_{region}\left(SL_{n},N\right)$$

$SL_{node}$ denotes the strategic node number and *N* shows the number of nodes from sparse region have $SL_{node}$ in their transmission range. When all members of a sparse region are in the transmission that location is picked to forward the data from the sensor nodes of a sparse. $SL_{node}$ re-establishes its data path after every 30 s with candidate nodes and transmit their sensed data after every 60 s. It moves randomly in a $S_{reg}$, however always at lower pressure level than the void region nodes because the objective is to minimize the number of transmission for transmitting data between the sender and the receiver.

The total energy consumption is computed according to Equation (8).

(8) $$\begin{array}{c} \begin{array}{c} ET_{i}=(E_{tx}\left(i\right)\times Dist_{srd}+E_{rx}\left(i\times Neighbors\left(i\right)\right)+EPM_{i} \end{array} \end{array}$$

where $ET_{i}$ denotes total energy consumed in data packet transmission from the source to the destination, $E_{tx}\left(i\right)$ shows the transmission energy, $E_{rx}\left(i\right)$ denotes energy reception, $Dist_{srd}$ is used to chose minimum distance from the source to the relay and the relay to the destination, $Neighbors_{i}$ shows the number of neighbor nodes received the data packet and $EPM_{i}$ is the energy consumed in path management after every 30 s.

### 3.4. Channel Model

We use binary phase shift keying (BPSK) as a modulation scheme because bandwidth in UWSNs is severely limited and BPSK allows data to be carried out efficiently on acoustic channel with given bandwidth. It is calculated as given in Equation (9) as follows:

(9) $$\begin{array}{c} \begin{array}{c} S_{0}=\left(\sqrt{2}E/T\right)(\cos\left(2\pi ft\right)\\ S_{1}=\left(\sqrt{2}E/T\right)(\cos\left(2\pi ft+\pi \right) \end{array} \end{array}$$

where $S_{0}$ denotes when *θ* is 0 and $S_{1}$ shows θ=180. *E* is energy, *T* shows time period and *f* is the frequency of the signal which is transmitted over a path of length *l*. Total attenuation due to spreading loss and absorption co-efficient on the basis of spreading is given as follows [25]:

(10) $$\alpha =\frac{10^{\alpha (f)}}{\left(10\right)}$$

(11) 10log(A(l,f))=k10log(l)+l10log(α(f))

It is physically impossible to design a noise free channel because whenever signal is broadcasted in the channel, it is affected by different noises like turbulence $\left(N_{tu}\right)$, wind $\left(N_{w}\right)$, thermal $\left(N_{th}\right)$, and shipping $\left(N_{s}\right)$. The total noise (TN(f)) calculated as shown in Equation (12).

(12) $$TN\left(f\right)=N_{tu}\left(f\right)+N_{w}\left(f\right)+N_{th}\left(f\right)+N_{s}\left(f\right)$$

where:

(13) $$10\log(N_{tu}\left(f\right))=17-30\log\left(f\right)$$

(14) $$10\log\left(N_{w}\left(f\right)\right)=50+7.5w^{1/2}+20\log\left(f\right)$$

(15) $$10\log(N_{th}\left(f\right))=-15+20\log\left(f\right)$$

(16) $$10\log(N_{s}\left(f\right))=40+20\left(s-0.5\right)+26\log\left(f\right)-60\log\left(f+0.03\right)$$

SNR of the received signals at the destination is calculated as follows in Equation (17):

(17) $$SNR=\frac{\mu }{\sigma }$$

where *μ* is the mean of the received signal with and without a noise $S_{1}$ by Equation (9) and *σ* is the standard deviation of the signal. $n_{01}$ denotes total number of received signals with and without noise at the destination. Mean is calculated as follows in Equation (18):

(18) $$\mu =\frac{\left(S_{0}+S_{1}\right)}{n_{01}}$$

where standard deviation is given below:

(19) $$\sigma =\sqrt{\frac{{\left(\mu -S_{0}\right)}^{2}+{\left(\mu -S_{1}\right)}^{2}}{n_{01}}}$$

We assume the additive white gaussian noise (AWGN) model because it counters ambient noises in a better way. The channel capacity *C* [26] with bandwidth *B* at time *T* is given as follows:

(20) $$C=B\frac{1}{2T}\ln\left(1+\frac{\mu }{\sigma }\right)$$

**Figure 7:**
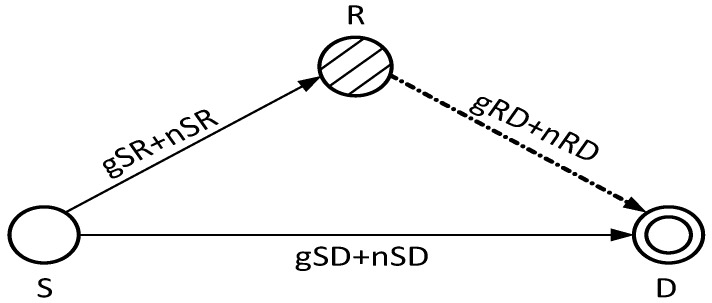
Cooperation.

Figure 7 shows the basic cooperation technique procedure, S transmits its data packet to the destination node D directly and via a relay node R. Here, the noise and the fading are denoted by *n* and *g*, respectively. The relationship between the transmitted and the received signals are given in Equations (21)–(23) [27]. The faded signal received at D directly from S is given as below:

(21) $$Y_{SD}=X_{S}g_{SD}+n_{SD}$$

Signals received at R, and from R to D are given as:

(22) $$Y_{SR}=X_{S}g_{SR}+n_{SR}$$

(23) $$Y_{RD}=Y_{SR}g_{RD}+n_{RD}$$

$X_{S}$ is the original signal generated and transmitted by S, $Y_{SD}$ is the signal received at D directly from S, $Y_{SR}$ is the received signal at R and $Y_{RD}$ is the signal received at D via R. $g_{SD}$, $g_{SR}$ and $g_{RD}$ denote channel gains over source to destination, source to relay and relay to destination links, respectively. $n_{SD}$ and $n_{SR}$ denote channel noises over source to destination and to relay links, respectively. $n_{RD}$ is the channel noise over relay to destination link.

At D, MRC technique is used to combine two different copies of the same signal, one received directly from S and the other via R. Thus the signal at D after applying MRC is given as [28]:

(24) $$Y_{D}=Y_{SD}+\frac{Y_{SR}Y_{RD}}{1+Y_{SR}+Y_{RD}}$$

### 3.5. Opportunistic Cooperation

**Figure 8:**
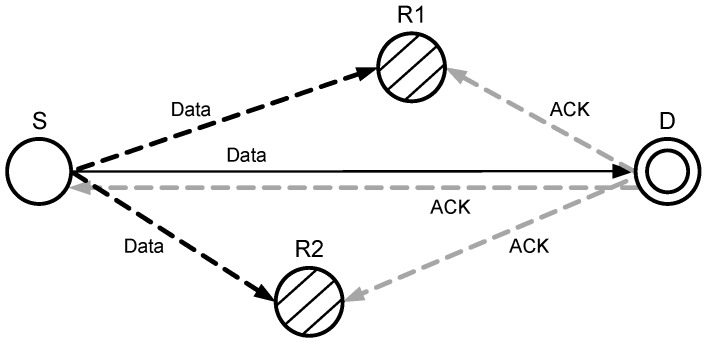
Data packet transmission without cooperation.

In Co-Hydrocast, fixed nodes are not involved in the minimization of the number of transmissions. However, in Co-Improved Hydrocast fixed nodes play a role in reducing the number of transmissions. The cooperation is performed in both schemes at each destination node to increase the SNR of the received signal. Details of the procedure are given below:

When a source node transmits a signal towards a destination node, it is overheard by other neighboring nodes, and they keep a copy of the received data packet. Destination node after receiving the source node signal, checks if SNR meets the threshold or not. If the SNR is (according to Equation (17)) within the acceptable range, then the destination node accepts the received signal and broadcasts an acknowledgment (ACK), as shown in Figure 8. After receiving the ACK, the relay nodes discard the stored copy of the source node signal.

**Figure 9:**
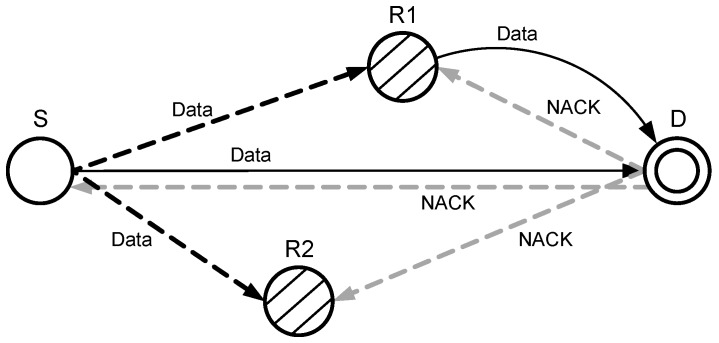
Data packet transmission with cooperation.

In sparse networks, the probability of data packet drop ratio and the low SNR at the destination increases. Figure 9 shows when the SNR at the D is less than the pre-defined threshold. In that case, a negative ACK (NACK) is generated and broadcasted by D, which is received by R1 and R2. Receiving NACK, a relay node of highest priority forwards the copy of the S towards D. Both copies of the signal that are received directly from a source node and via relay node are combined at the destination node by MRC technique to avoid the re-transmission.

### 3.6. Minimization of Energy Consumption

In order to design an energy efficient routing protocol, linear programming is one of the most commonly used mathematical techniques. It begins with a problem formulation of an objective function that has some linear constraints. In this subsection, we will discuss how linear programming based model reduces the energy consumption resulting in maximum network lifetime.

In our proposed schemes, sensor nodes consume their energy in data communication between the source and the destination while receiving and transmitting a data packet. Therefore, based on this assumption, we have formulated the objective function that is given in Equation (25).

(25) $$Min\sum_{r=1}^{r=max}E_{consumed}\left(r\right)\;\;\forall \;r\in R$$

where $E_{consumed}$ is the energy consumed by the sensor node during the data communication that is computed as given in Equation (26).

(26) $$E_{consumed}=\sum_{s=1}^{d=n}D\left(i\right)\times \left(E_{tx}+E_{rx}\right)$$

$E_{tx}$ is a maximum transmission energy over distance D, $E_{rx}$ shows a receiving energy over D. These are calculated as given in Equations (27) and (28).

(27) $$E_{max-tx}=P_{tx}\left(DPS/DR\right)$$

where DPS denotes data packet size, DR is a data rate, $P_{tx}$ represents a transmission power and $P_{rx}$ is a receiving power.

(28) $$E_{max-rx}=P_{rx}\left(DPS/DR\right)$$

such as,

(29) $$E_{tx,rx}\leq E_{i}^{max}\;\;\forall \;i\in N\;\;\;$$

(30) $$E_{tx,rx}\geq E_{i}^{min}\;\;\forall \;i\in N\;\;\;$$

(31) $$SNR\geq SNR_{th}\;\;\forall \;SNR\in N\;\;\;$$

(32) $$D_{s}^{d}\leq D_{s}^{dmax}\;\;\forall \;s,d\in N\;\;\;$$

(33) $$D_{s}^{d}\geq D_{s}^{dmin}\;\;\forall \;s,d\in N\;\;\;$$

According to Equation (25), our objective is to minimize the energy consumption in each round. Such that the summation of all rounds resulting in high network lifetime. Equation (26) gives the explanation about the type of energy consumed in each round and Equations (27) and (28) explain how the transmission and the receiving energy in calculated at each hop between the source and the destination. Constraints defined in Equations (29) and (30) ensure that energy must be within these constraints to receive and transmit a data packet in any round successfully. Equation (31) constraints ensure that each packet received at the destination must satisfy the predefined SNR threshold otherwise it will perform the cooperation to enhance the SNR to increase the reliability of the data packet. Similarly, Equations (32) and (33) are used to restrict the transmission of data communication over too long or too small distances in order to keep data packets within the transmission range of a node and reduce the number of hops for conserving nodes energy for a long time. Suppose the total available energy is $\left(EN_{total}\right)$, that is computed as given below:

(34) $$EN_{total}=E_{initial}\times N$$

$EN_{initial}$ denotes the initial energy of a single nod at the time of deployment and *N* shows the number of nodes in the network.

The total $E_{consumed}$ after round (r) is calculated as shown in Equation (35).

(35) $$E_{consumed}=\sum_{r=1}^{r=r}\left(EN_{total}\left(r\right)-E_{consumed}\left(r\right)\right)$$

Let D be divided in to K number of levels then the minimum energy consumption over K be computed as given in Equations (36) and (37).

(36) $$E_{min-tx}=E_{tx}/K$$

(37) $$E_{min-rx}=E_{rx}/K$$

Here, it can be noted that the energy utilization during data transmission and data collection over D provides the encouraging results in response to the objective function given in Equation (25).

**Graphical analysis:** Let we have a scenario in which transmission range (250 m) be divided in to *K* levels i.e., K=1,2,3,4, and 5. Where N=100, DPS=200B, DR=10 Kbps, $(P_{tx}$, and $P_{rx})$ is 2watt (W) and 0.1 W, respectively. From these values, using Equation (27) $E_{tx}$ is 0.32 J for K=1 and from Equation (36), $E_{tx}=0.064$ J for K=5. By using Equation (28), $E_{rx}=0.016$ J and from Equation (37), $E_{rx}=0.0032$ J for K=1 and K=5, respectively. Equations (26)–(28), could be denoted as given in Equations (38)–(40).

(38) $$0.0672\leq E_{tx}+E_{rx}\leq 0.336$$

(39) $$0.064\leq E_{tx}\leq 0.32$$

(40) $$0.0032\leq E_{rx}\leq 0.016$$

**Figure 10:**
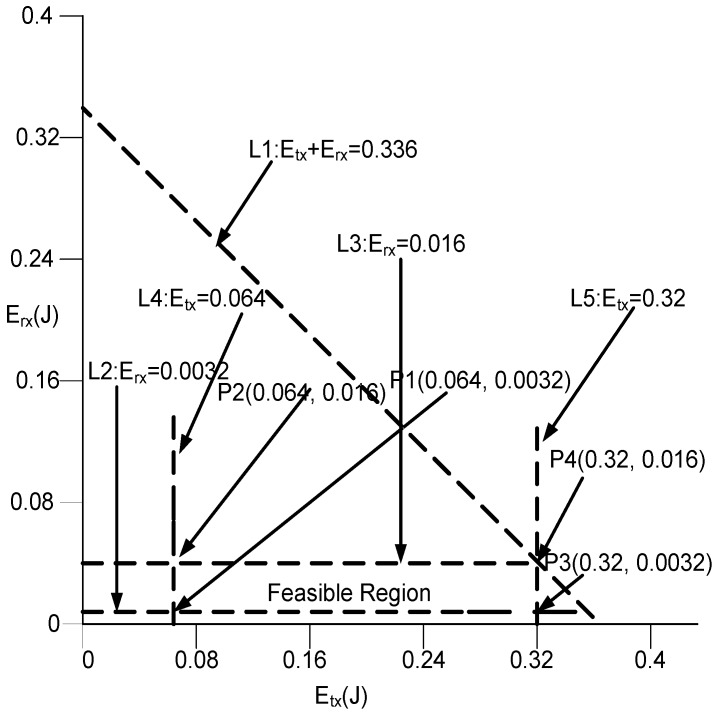
Feasible Region of Energy Minimization.

By keeping in mind, bounds given by Equations (38)–(40); Figure 10 shows the intersection of lines (L1, L2, L3, L4, and L5) that contains the set of all feasible solutions. This region known as feasible region. The solution of each point (P) is valid in this region. Now, we test every vertex depicted in Figure 10 as:

at $P_{1}$: 0.064+0.0032=0.0672 J,
at $P_{2}$: 0.064+0.016=0.08 J,
at $P_{3}$: 0.32+0.0032=0.332 J,
and at $P_{4}$: 0.32+0.016=0.336 J.

Therefore, it is proved that all calculated solutions are valid. The energy consumption will be minimum with in the given premises of the feasible region resulting in high network lifetime.

### 3.7. Throughput Maximization

In this section, we have formulated an objective function to maximize the network throughput, the purpose of this model is to show that the minimization of energy consumption did not compromise the throughput of the network in our proposed schemes is comparatively still better than the compared existing scheme (Hydrocast).

In our proposed schemes (Co-Hydrocast, improved-Hydrocast, and Co-improved Hydrocast), data packets are received via cooperative routing or with out cooperation. The network throughput is, the total number of data packets received successfully at the sink. The SNR check is performed at each link to ensure that data packets arrived the destination are within the SNR, if the data packet received at the destination have SNR within the predefined threshold then the data packet is considered in throughput. Therefore, based on these considerations, an objective function is formulated that is given in Equation (41).

(41) $$Max\sum_{r=1}^{r=max}TP\left(r\right)\;\;\forall \;r\in R$$

where TP(r) denotes total number of data packets received in r (rounds) and TP is computed as given in Equation (42). It shows number of packets (P) those meet the $SNR_{th}$.

(42) $$TP=\sum_{P=1}^{P_{max}}SNR\times P\;\;\forall \;P\in TP$$

Equation (43) shows that a packet is only counted as a successful transmission when SNR of a signal is 1, if the received signal SNR is not within the threshold value then TP=(0×P).

(43) $$\begin{array}{c} SNR=\left\{\begin{array}{cc} 1 & \mathrm{if}\;SNR\geq {SNR}_{th}\\ 0 & \mathrm{if}\;SNR<{SNR}_{th} \end{array}\right. \end{array}$$

such that;

(44) $$E_{tx,rx}\leq E_{i}^{max}\;\;\forall \;i\in N\;\;\;$$

(45) $$E_{\left(tx,rx\right)}\geq E_{\left(i\right)}^{min}\;\;\forall \;i\in N\;\;\;$$

(46) $$SNR\geq SNR_{th}\;\;\forall \;SNR\in N\;\;\;$$

(47) $$D_{s}^{d}\leq D_{s}^{d_{max}}\;\;\forall \;s,d\in N\;\;\;$$

(48) $$D_{s}^{d}\leq D_{s}^{d_{min}}\;\;\forall \;s,d\in N\;\;\;$$

(49) $$Max\sum_{CN=1}^{CN_{max}}CN_{B}\;\;\forall \;CN_{B}\in N\;\;\;$$

(50) $$Min\sum_{NC=1}^{NC_{max}}NC_{B}\;\;\forall \;NC_{B}\in N\;\;\;$$

Our main objective is to maximize the network throughput (that is Equation (41)) by meeting all the given constraints. Equations (42) and (43) ensures that all packets received at the destination are meeting the predefined SNR threshold ($SNR_{th}$) will be accounted as a successful data packet. Constraints defined in Equations (44) and (45), make that $E_{tx},E_{rx}$ should not exceed the maximum and the minimum limits. These restrictions are set to avoid the wastage of energy. SNR constraint is defined in Equation (46) to ensure the reception of error free packets at the sink and to conserve the energy and avoid a packet loss over long distances and minimize the number of hops for maximizing network lifetime and reducing propagation delay (Equations (47) and (48) are defined). Similarly, to avoid buffer overflow of sensor nodes, two constraints Equation (49) and Equation (50) are used to allocate a desired bandwidth as per requirement.

Let suppose, the total available bandwidth (KHz) be $T_{B}$, such that,

(51) $$T_{B}=CN_{B}+NC_{B}$$

where $CN_{B}$ and $NC_{B}$ denote cooperative nodes bandwidth and non cooperative nodes bandwidth, respectively. $CN_{B}$ is computed as follows:

(52) $$CN_{B}=T_{B}\left(i\right)\times {\left(N_{n}\right)}^{p}\;\;\forall \;i\in N$$

$T_{B}\left(i\right)$ is the total available bandwidth for a cooperative node i, $N_{n}$ number of nodes present in its transmission range for the relay cooperation, $C_{N}$ represents the number of times the cooperation is performed, $NC_{B}$ is the total available bandwidth for non cooperative nodes, and p=[0,1] is a probability of relay node existence.

(53) $$CN_{B}=CN_{B}\times C_{N}$$

(54) $$NC_{B}=T_{B}-CN_{B}$$

Here, it can be noted that the bandwidth allocation during data collection provides the encouraging results in the response to the objective function given in Equation (25).

**Graphical analysis**: Let suppose, we have a scenario in which $T_{B}=282$ KHz to 300 KHz, N=100, $N_{n}=3$, and $C_{N}=3$. We have calculated the value of $CN_{B}$ by using the values of *N*, $T_{B}$, $N_{n}$, and $CN_{N}$. $CN_{B}=9$ KHz when p=0 and 18 KHz at p=1. Similarly, $NC_{B}$ is computed by using Equation (54). Equations (51), (53), and (54), could be denoted as given in Equations (55)–(57) (with units KHz).

(55) $$9\leq CN_{B}\leq 18$$

(56) $$282\leq NC_{B}\leq 291$$

(57) $$291\leq CN_{B}+NC_{B}\leq 300$$

**Figure 11:**
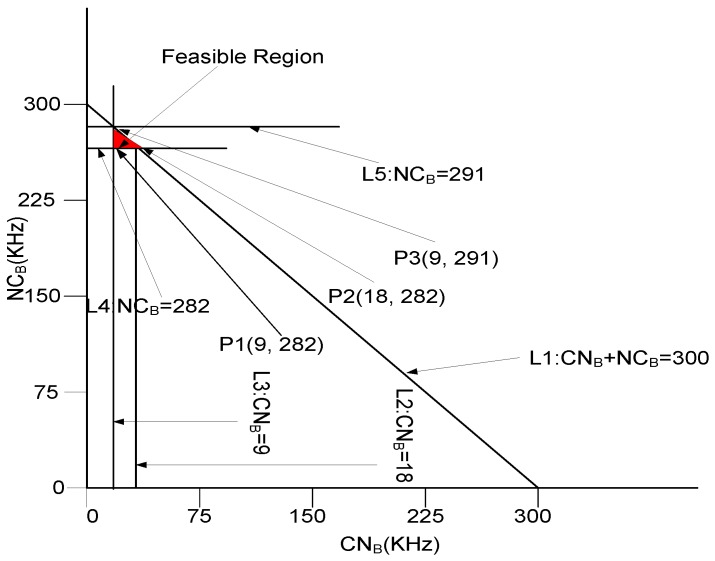
Feasible Region for Throughput Maximization.

The bounds provided by Equations (55)–(57) are shown in Figure 11. The intersection of lines L1, L2, L3, L4, and L5 shows the bounded region of all feasible solutions. Each point in region gives a valid solution and each vertex of feasible region is tested that depicted by Figure 11.

at $P_{1}$: 9+282=291 KHz,
at $P_{2}$: 18+282=300 KHz, and
at $P_{3}$: 9+291=300 KHz,

Hence, it is proved that all points are valid in the depicted feasible region. Therefore, a desired bandwidth can be easily allocated at any point within the depicted premises of the feasible region as per requirement.

## 4. Simulations and Results

In simulations, we evaluate our proposed schemes by comparing them with an existing scheme Hydrocast [4]. The Hydrocast scheme is efficient in dense network however in sparse conditions lengthy path establishment increases the path loss probability resulting in high packet drop ratio. Therefore, the number of re-transmissions increases in the network which leads to more energy consumption and high propagation delay. Simulation results show that our proposed schemes outperform the compared existing scheme.

This section consists of two subsections. In Section 4.1, simulation results are discussed for fast (7.71 m/s) moving AUVs in the network. Section 4.2 includes performance discussion of the proposed schemes when slow moving (0.3 m/s) sensor nodes are deployed at strategic locations in the network.

### 4.1. AUVs

The simulation parameters [4] are given in Table 2. AUVs varies from 30 to 100, are deployed in a three dimensional network of 5 km × 5 km × 5 km. AUVs move independently with maximum speed of 15 knots (7.716 m/s). The transmission range of AUV is set 1000 m and twelve AUVs are deployed at strategic locations in the network while rest of AUVs are randomly deployed in the network. Each AUV transmits its sensed data to the destination after every 60 s.

**Figure 12:**
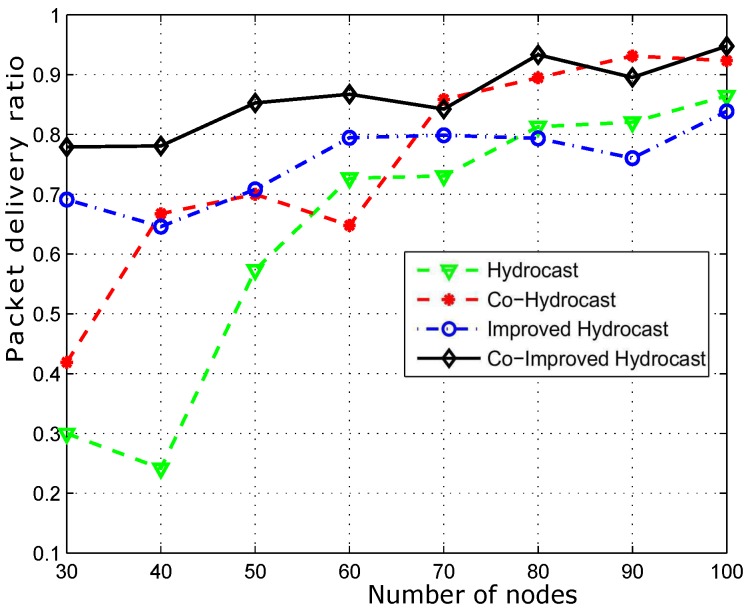
Successfully transmitted data packets to sink.

Figure 12 shows the packet delivery ratio. Hydrocast has low throughput when the network density is low. The reason is that the breakage of established recovery paths for void nodes due to the mobility of sensor nodes. In sparse network conditions, more void nodes are found that need recovery paths for the data packet transmission. As the network density increases, the number of void nodes decreases, and ultimately less number of recovery paths are established in the network and vice versa. Improved Hydrocast shows better performance due to the deployment of nodes at the strategic locations in the network. Co-Hydrocast also has better results than the Hydrocast due to the cooperation technique in Co-Hydrocast. Whenever, a data packet fails to successfully reach the destination, or the received signal SNR according to Equation (17) is lower than the threshold, MRC technique is used to combine signals for avoiding re-transmissions. Co-Improved Hydrocast outperforms all compared schemes, and has the highest packet delivery ratio. It takes the advantage of both opportunistic cooperation and strategic location mechanism to improve the PDR of the network nodes.

**Figure 13:**
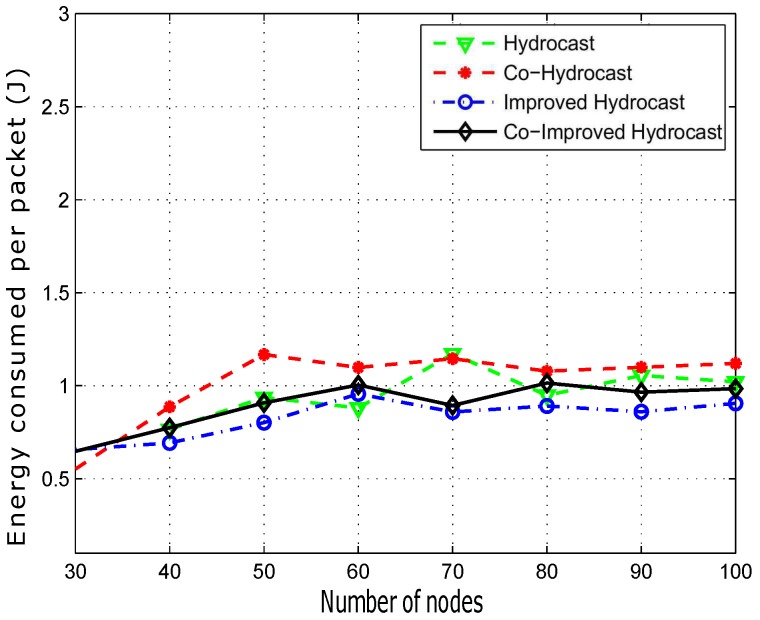
Average end to end energy consumption.

In Figure 13, the average energy consumption of the successfully delivered packets is shown. Improved Hydrocast has low energy consumption due to the establishment of short recovery path for void nodes. The strategic deployment of sensor nodes in the Improved Hydrocast played an important role in reducing the length of recovery paths in the sparse network conditions. Co-Improved Hydrocast scheme consumes more energy than the Improved Hydrocast due to the opportunistic cooperation technique that is implemented to reduce the packet drop ratio in the network. In cooperation, the relay node also forwards the packet to the destination which results in more energy consumption. In Hydrocast, the energy consumption for each data packet delivery is higher than the other two proposed schemes. Recovery paths for void nodes in Hydrocast are lengthy in sparse network conditions, due to which more energy is consumed to deliver the data packet to the destination. Co-Hydrocast consumes more energy to deliver data packets because of cooperation technique and lengthy recovery paths for void nodes in sparse network condition. Both Co-Hydrocast and Co-Improved Hydrocast uses cooperation technique for reducing packet drop ratio however the deployment of sensor nodes at the strategic locations enabled void nodes to establish a short path for the data packet transmission to the destination.

**Figure 14:**
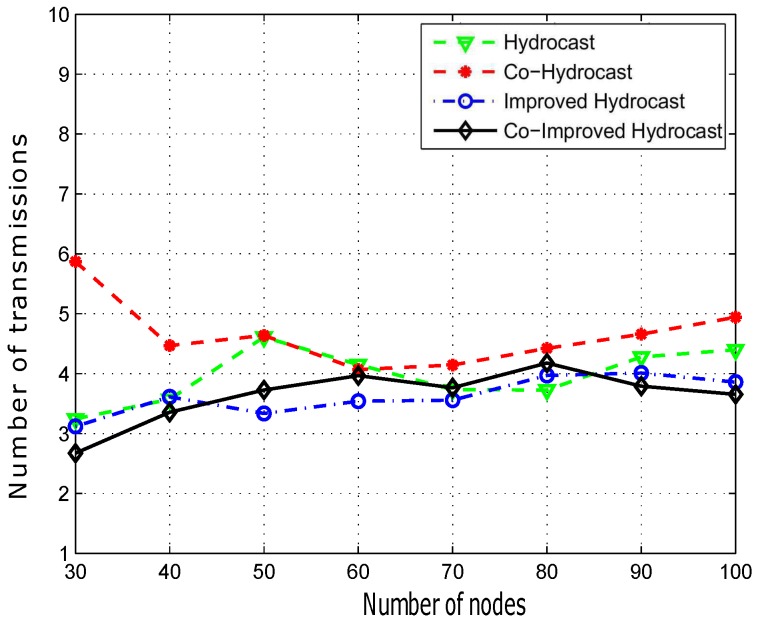
Number of transmissions for packet delivery.

Figure 14 determines the average number of transmissions to deliver a data packet successfully to the destination. Hydrocast on average, deliver a data packet in more number of transmissions than the Improved Hydrocast and the Co-Improved Hydrocast because of lengthy recovery paths for void nodes in the sparse networks. On average Hydrocast makes 3.1 transmissions for delivering a data packet to the sinks deployed at the surface. Whereas, with the increase of nodes, the number of transmissions decreases that shows that Hydrocast performs better in the sparse deployment. In our proposed schemes, the deployment of sensor nodes at strategic locations in the network, not only reduce the long recovery paths when the network density is low but also help some other candidate nodes to transmit their data to the destination in less number of transmissions. The number of hops are less in our schemes because they use AUVs to gather data from sensor nodes. Average number of hops in Improved Hydrocast are 3.05 which is slightly better than Hydrocast. Co-Improved Hydrocast also uses strategically deployed nodes, but due to opportunistic cooperation, the average number of transmissions for a data packet delivery slightly lower than the Improved Hydrocast, Co-Hydrocast, and Hydrocast. When network is sparse, its average number of hops are less (2.8 transmissions) which increases as the density increases because it starts establishing shorter routing paths to conserve the network energy however still less than the other compared schemes.

**Figure 15:**
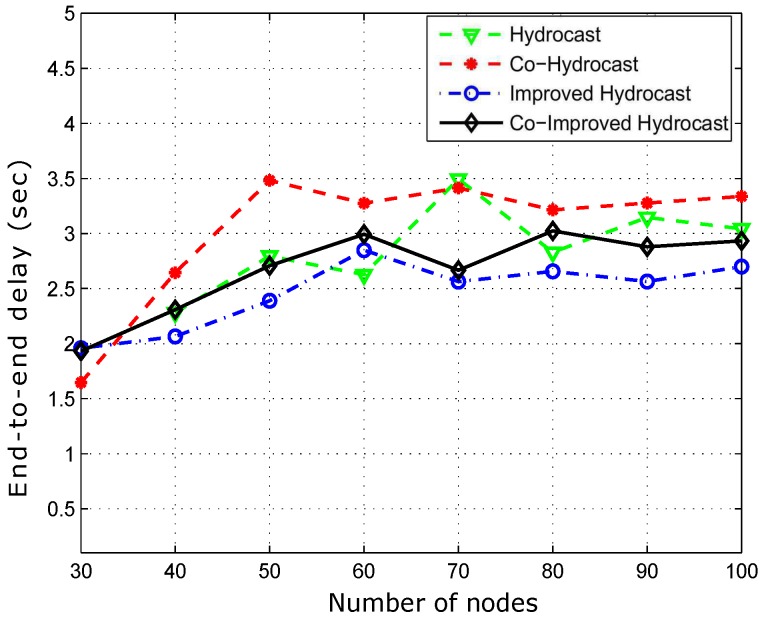
Average propagation delay between source and destination.

The average propagation delay is plotted in Figure 15. Co-Hydrocast has higher latency than Hydrocast due to cooperative transmission, while both suffer from lengthy recovery paths issue in sparse networks which yields high average delay. In Hydrocast, when the network is sparse, the average propagational delay is 2.25 s and in Co-Hydrocast it is 2.6 s due to relay cooperation in order to obtain signals within the predefined SNR. As the network density increases, the average propagational delay also increases due to increase in number of collisions that results in path loss and increases the delay. Improved Hydrocast outperforms the compared scheme and has lower propagation delay (2.10 s) due to short path from the source to the destination with the help of strategic deployment of nodes. Its delay increases due to short paths that increase the number of hops between source and the destination resulting in more delay however, it has lowest delay amongst all the compared existing scheme and proposed schemes. Co-Improved Hydrocast also takes advantage of the strategic locations however due to cooperation, it has slightly higher average propagation delay than Improved Hydrocast scheme. Cooperative techniques show improvement in the throughput, however at the cost of propagation delay as shown in Figure 15.

### 4.2. Simple Sensor Nodes

The simulation parameters [4] are; three dimensional network of size 1 km × 1 km × 1 km is used. Different number of nodes varying from 120 to 300, are deployed randomly in the network. These nodes are not static, and move with the speed of 0.3 m/s. Transmission range is 250 m for these nodes. Nodes move independently, and after every 60 s, they forward their sensed data towards the destination.

**Figure 16:**
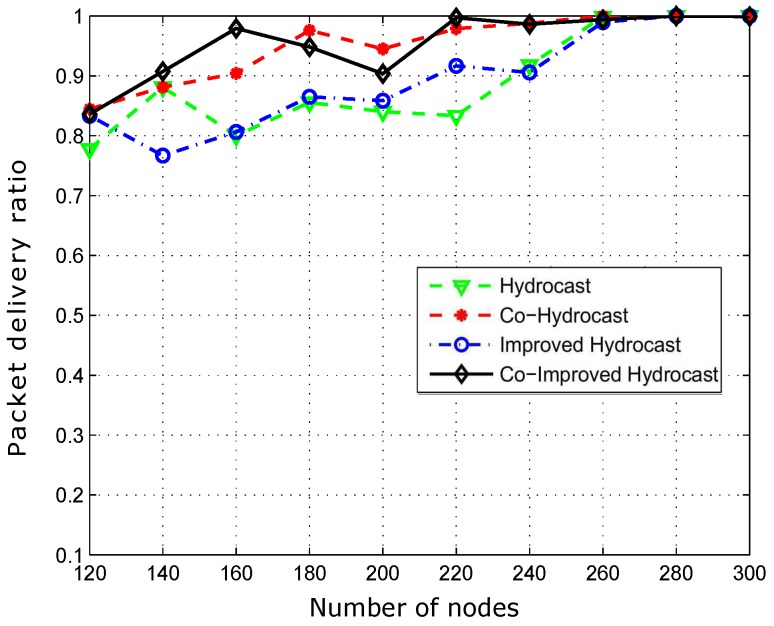
Successfully transmitted data packets to sink.

Figure 16 shows packet delivery ratio (PDR). Hydrocast has lower throughput than the other proposed scheme due to fast mobility of nodes which leads to path loss. However, the PDR improved in dense networks as shown in Figure 16. Improved-Hydrocast have shorter routing paths to overcome path loss due to the mobility of sensor nodes which minimize void regions with strategic deployment of nodes. Co-Hydrocast and Co-Improved Hydrocast has higher PDR due to cooperation technique, a data packet that has high BER is not discarded at the destination but it is combined with the copy of same signal received from the relay node using MRC technique. Cooperation technique improves the throughput, and reduces the packet drop ratio in the network at the cost of propagation delay and energy consumption.

**Figure 17:**
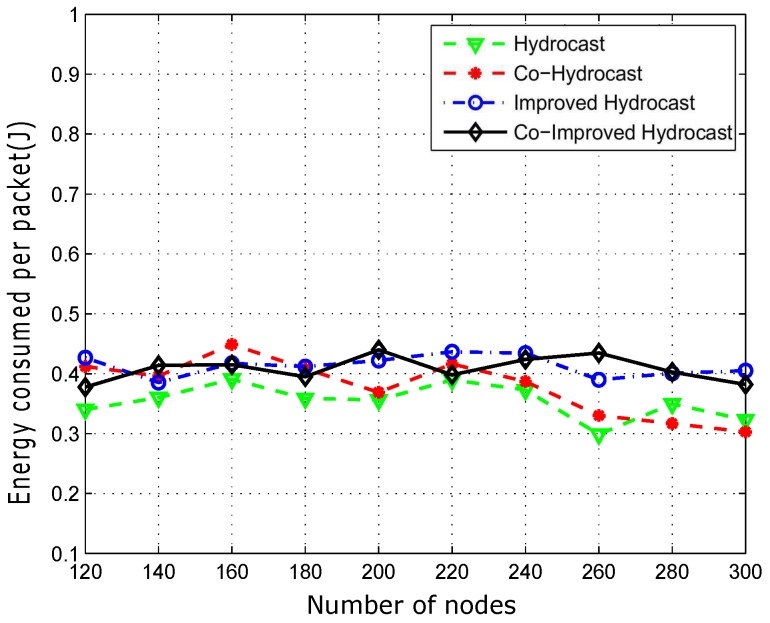
Average energy consumption between source and destination.

Average energy consumption of successfully transmitted data packets is plotted in Figure 17. Hydrocast consumes less energy for a data packet delivery when the network is denser due to less number of void nodes, short recovery paths and no cooperation. Co-Hydrocast and Co-Improved Hydrocast schemes have high energy consumption due to opportunistic cooperation technique which has influential factor of redundant transmissions between the source and the destination which leads to high energy consumption in dense networks as shown in Figure 17.

**Figure 18:**
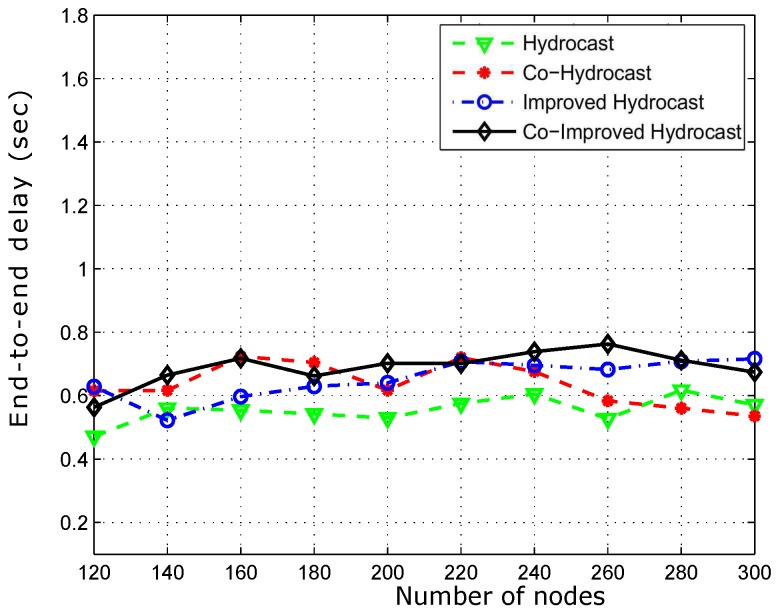
Average propagation delay between source and destination.

Figure 18 shows the average propagation delay of the proposed schemes. Co-Hydrocast has high delay than Co-Hydrocast due to opportunistic cooperation while both suffer from lengthy routing paths. Hydrocast has more propagational delay due to lengthy recovery paths from void regions. As shown in Figure 18 that the delay is minimum in our proposed schemes due to short paths in the sparse networks.

**Figure 19:**
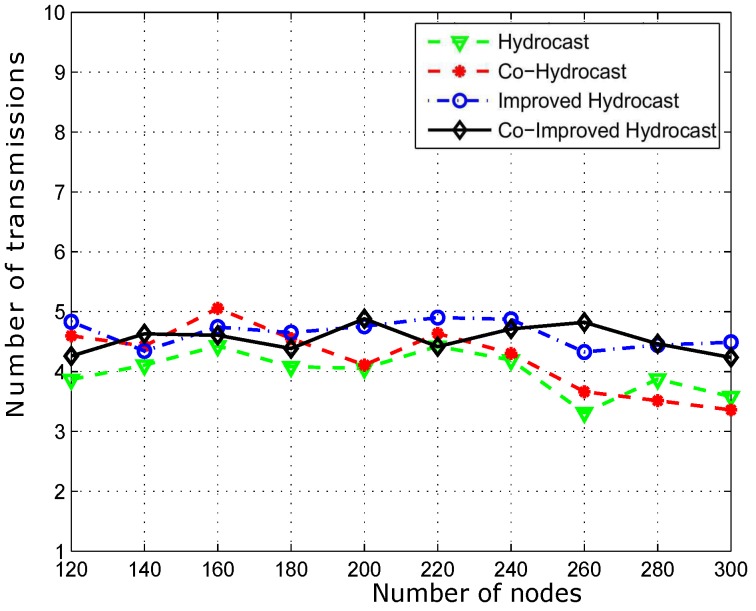
Number of transmissions for data packet delivery.

Figure 19 shows the successfully transmitted data packets from a source to a destination. In dense networks, PDR of proposed techniques decreased due to the selection of a relay node which has at least one neighbor as forwarder to avoid a void node selection. However, cooperation technique improved PDR due to the deployment of sensor nodes at strategic locations.

## 5. Performance Trade-Offs

Hydrocast achieves high packet delivery ratio by establishing lengthy recovery paths resulting in high end to end delay. Improved Hydrocast fixes the high end to end delay issues by deploying nodes at strategic locations in the network. Co-Hydrocast achieves the high throughput via opportunistic cooperation technique, however, at the cost of high energy consumption due to involvement of relay nodes in packet forwarding. Co-Improved Hydrocast uses both opportunistic cooperation technique and strategic location based node placement to achieve high throughput and low latency. The performance trade-offs of the simulated protocols are summarized in Table 3.

## 6. Conclusions and Future Work

In this paper, Co-Hydrocast, Improved Hydrocast and Co-Improved Hydrocast routing protocols are proposed for UWSNs. Major limitations of Hydrocast were lengthy recovery paths in sparse networks which increased the packet drop ratio and the energy consumption of the network. Energy wasted by every dropped packet is minimized by establishing small routing paths between the source and the destination to ensure a reliable delivery of the data packet. The deployment of nodes at strategic locations helped in selecting an efficient relay node resulting in less energy consumption even in the sparse networks which leads to high PDR. Co-operative opportunistic (Co-Hydrocast, Co-Improved Hydrocast) routing minimized the packet drop ratio by using MRC technique to enhance the PDR at the cost of high energy consumption and more propagation delay. Simulation results reveal that our proposed schemes outperformed the existing compared counterpart scheme in terms of energy consumption per packet and PDR. Improved Hydrocast has minimized the propagation delay and Co-operative schemes consumed more energy which is compensated with high throughput.

As a future work, we have planned to investigate the impact of nodes mobility due to water currents and interference on the network lifetime. The earlier issue will be handled by incorporating a mobility model and for later issue some technique will be proposed. Then influence of both issues will be analyzed in terms of network lifespan, end to end delay and PDR. 

## Figures and Tables

**Table 1:** State of the art related work.

Protocol	Feature	Achievements	Limitation
GEDAR [2]	Geo-opportunistic routing protocol.	Avoid void hole occurrence, recover void holes, high network throughput.	High end to end delay and energy consumption during node recovery from void hole by depth adjustment mechanism.
Hydrocast [4]	Pressure based routing for UWSNs.	Utilize the duplicate packets transmission of opportunistic routing to recover data from void nodes.	Perform efficiently in dense networks.
Cooperative diversity with incremental best relay technique [5]	Incremental best relay cooperation on demand.	Saves channel resources and low processing overhead.	High packet drop ratio and low network lifetime.
VARP [6]	Void aware depth based routing.	Reduction in recovery fullbacks, efficient handling of node mobility and reduction in packet drop ratio.	High energy consumption and end to end delay.
Energy efficient cooperative communication for data transmission [7]	Cooperative communication.	Improvement in packet transmission in lossy network.	Low performance in sparse network conditions.
DBR [8]	Depth based routing.	High packet delivery ratio in dense network.	High load on low depth nodes, redundant packet transmissions.
EEDBR [9]	Depth and residual energy based routing.	Energy balancing and improvement in packet delivery ratio.	Redundant packet transmission and high energy consumption in denser networks.
WDFAD-DBR [10]	Depth based routing.	Void hole avoidance in order to minimize energy consumption.	Finding 2 hop neighbors does not eliminate the occurrence of void hole
H2-DAB [11]	Multi hop dynamic addressing based routing.	High data packet delivery ratio and prolonged network lifetime.	High latency, high load on low depth nodes and processing overhead.
AURP [12]	Use of AUVs as relay nodes.	Improvement in data packet delivery and reduction in energy consumption.	High control messages exchange overhead.
ERP2R [13]	Residual energy and distance based routing.	Low latency and energy efficiency.	Redundant packet transmissions and low performance with increased mobility of nodes.
DEADS [14]	Sink mobility, single and multiple relay cooperative routing.	Increase in throughput and reduction in packet drop ratio.	High energy consumption and high control messages exchange overhead.
Energy and time efficiency in depth based routing [15]	Time of arrival (ToA) ranging technique.	Reduction in energy consumption from multi path forwarding redundancy.	High end to end latency.
Joint cooperative routing and power allocation [16]	Cross layer cooperative routing	Relatively lower collision probability compared to other cooperative schemes.	Not applicable for time critical applications.
Delay and lifetime performance [17]	Routing using mobile sink.	Increment in network lifetime and packet delivery ratio.	No time bound for data transfer in case of random mobility of sink resulting in mobile sink buffer overflow.
BTM [18]	Optimum transmission distance to the sink	Improvement in network lifetime, balanced energy consumption	Transmission loop formation and high energy consumption in case of direct transmission over long distances.
Energy efficient cooperative communication [19]	Clustering and cooperative communication	Energy efficiency relative to direct transmissions.	High energy consumption in cluster formation.
Enhanced energy balanced data transmission [20]	Balanced energy transmission.	Improvement in network life time, high throughput and low packet drop ratio.	Throughput and network lifetime decreased with increased network radius.

**Table 2:** Simulation Parameters.

Simulation Parameters	Values
Network Dimension for Nodes	1 km × 1 km ×1 km
Transmission Range of Node	250 m
Speed of nodes	0.3 m/s
Network Dimension for AUV	5 km × 5 km × 5 km
Transmission Range of AUV	1000 m
Number of AUVs	30 to 100
Speed of AUV	7.716 m/s
AUV transmit sensed data after	60 s
AUV re-establish path after	30 s

**Table 3:** Performance trade-offs made by the protocols.

Protocol	Achieved Parameter	Compromised Parameter
Hydrocast	Packet delivery ratio (Figure 16)	Propagation delay (Figure 15)
Improved Hydrocast	Number of transmissions (Figure 14)	Nodes placement overhead (Figure 2b)
Improved Hydrocast	Propagation delay (Figure 15)	Nodes placement overhead (Figure 2b)
Co-Hydrocast	Packet delivery ratio (Figure 12)	Energy consumption (Figure 13)
Co-Hydrocast	Packet delivery ratio (Figure 12)	Number of transmissions (Figure 14)
Co-Improved Hydrocast	Propagation delay (Figure 15)	Nodes placement overhead (Figure 2b)
Co-Improved Hydrocast	Packet delivery ratio (Figure 12)	Energy consumption (Figure 13)
